# Exploring possible causes of fatal burns in 2007 using Haddon’s Matrix: a qualitative study

**DOI:** 10.5249/jivr.v7i1.501

**Published:** 2015-01

**Authors:** Homayoun Sadeghi-Bazargani, Saber Azami-Aghdash, Shahnam Arshi, Mirkazem Mohammad Hosseini, Bahram Samadirad, Mehryar Nadir Mohammadi, Amin Daemi, Reza Mohammadi

**Affiliations:** ^*a*^Road Traffic Injury Research Center, Tabriz University of Medical Sciences, Tabriz, Iran.; ^*b*^WHO Collaborating Center on Community Safety Promotion, Karolinska Institute, Stockholm, Sweden.; ^*c*^Tabriz Health Services Management Research Center, Faculty of Management and Medical Informatics, Tabriz University of Medical Sciences, Tabriz, Iran.; ^*d*^Department of Public Health Sciences, Shahid Beheshti University of Medical Sciences, Tehran, Iran.; ^*e*^Meshkinshahr Health Network, Ardabil University of Medical Sciences, Ardabil, Iran.; ^*f*^Research Center of Forensic Medicine, Iranian Legal Medicine Organization, Tehran, Iran.

**Keywords:** Fatal burns, Haddon’s Matrix, Injury, Qualitative study

## Abstract

**Background:**

Burns are a major factor in injury mortality. The aim of this study was to explore the possible causes of fatal burns using Haddon’s Matrix.

**Methods:**

This is a qualitative study using a phenomenological approach. We collected elicitation interview data using nine corroborators who were the most knowledgeable about the index burn event. Immediately after recording, the data was verbatim. Each event was analyzed using Haddon’s Matrix.

**Results:**

Interviewees provided detailed information about 11 burn cases. Overall, 202 burn-related factors were extracted. Using Haddon’s Matrix, 43 risk factors were identified. The most common included the lack of basic knowledge of burn care, the use of unsafe appliances including kerosene heaters and stoves in hazardous environments such kitchens and bathrooms, poor burn care delivery system in hospitals, poor and unsafe living conditions, financial issues, and other factors detailed in the article.

**Conclusions:**

Our findings suggest burn related prevention efforts should focus on improving human living conditions, promoting the use of safe heating appliances, providing public burn-safety precautions education, and improving the quality of care in burn centers and hospitals. The use of Haddon’s Matrix in future injury research is discussed.

## Introduction

Burn injury is a serious, life-threatening problem and has terrible physical, psychological and financial effects on patients and society. ^1,2^ The World Health Organization (WHO) has reported that 238,000 individuals died of fire-related burns in 2000.^3^ Burn prevalence and mortality varies significantly between countries. ^4-6^ In Iran and other Eastern Mediterranean countries burns are a major public health problem. The prevention of burns requires adequate knowledge of the epidemiological characteristics of the injury event. ^7,8^

Injury epidemiology is defined as “the study of the distribution and determinants of injuries and safety in specified populations, and the application of this knowledge to prevent injuries and promote safety”.^9^ To develop preventive programs, it is important to have adequate information about the risk factors that affect the occurrence of burn injuries. The prevention of such injuries maybe examined with respect to the irepidemiology, biomechanics and behavioral science.

William Haddon had worked for many years on road safety in the USA. In 1970, he presented Haddon’s Matrix to the world of injury prevention. The most common form of the Haddon’s Matrix has four columns and three rows. Each row frames the timing of injuries as Pre-event phase, Event phase and Post-event phase. Each columns determines predisposing or enabling factors related to the injury occurrence.^10,11^ Haddon’s Matrix, however, is mainly a tool for qualitative study, ^12^ and there is a paucity of studies that focus on factors that increase the risk of fatality in burn events. We aimed in this study to explore the possible causes of fatal burns in Ardabil Provincial Burn Center using Haddon’s Matrix.

## Methods

A qualitative study was conducted in 2007 to explore and understand the possible causes of fatal burns using Haddon’s Matrix. Profile information and home addresses of fatal burn victims were retrieved from the hospital files of Ardabil Provincial Burn Center in 2007. The phenomenological approach was chosen to allow exploration of the conscious process experienced from the first-person point of view.^13,14^ A combination of convenience and purposive sampling methods were conducted to select the study participants. 

Purposive sampling is a method used in qualitative research to recruit participants who are best suited to provide the richest and maximum amount of information. This method helps to increase the validity of the information obtained because participants are able to provide a better account of their specific experiences and knowledge.^15,16^ Participants were included in the study if they had firsthand knowledge about the burn event.

Sampling was continued until data saturation was reached and no new information was obtained from the interviews. Haddon’s Matrix was used to improve the data saturation and analysis.^12,17^ Data were collected by qualitative non-structured interviews using open-ended questions. The study participants were encouraged to speak freely about the topics of interest. ^18^ Interviews were performed by two of the study researchers in the participant’s residence. Each interview lasted 30 to 45 minutes. In addition to taking fields notes, conversations were also recorded with participant's consent. Data was transcribed verbatim shortly after each interview, and were read and reviewed several times by the study team for immersion in obtained data. For each event a full description was written and analyzed by the two investigators using qualitative content analysis.

Content analysis consisted of the following process:

1-Familiarization with the data (reading and re-reading the data).

2- Generating primary codes (collating data relevant to the primary code).

3- Searching for themes (transfer primary codes into potential themes).

4- Formulating/ reviewing themes (generating a thematic “map”).

5- Defining and naming themes (generating clear definitions of themes).

6- Summarizing and positioning the themes into Haddon’s Matrix (“clustering” the final opportunity for analysis).

7- Assessing the reliability of analysis by two researchers in order to reach full agreement.

A single Haddon’s Matrix was formed for each event. An inclusive Haddon’s Matrix of causes and contributing factors in burn mortality was completed by integrating and summarizing the individual Haddon’s Matrices. Columns are identified using four injury mechanisms:

1- Human (also named as host) column.

2- Appliance (also named as vector, vehicle, object, equipment, etc.) column.

3- Physical environment column.

4- Social(economic) environment column.

Terminology for rows and columns headings depends on the type of injury. For example we have used the word “appliance” in burn studies as the second column heading, while “vehicle” would have been the most appropriate in examining traffic injuries.

The study was approved by the research committee of Ardabil University of Medical Sciences as part of a large epidemiological injury study. The study objectives were described before enrolling participants in the study. Potential participants were reminded that their participation is voluntary, and that their anonymity is protected. They were also advised that they were free to stop participation at any time. Through this process informed consent was obtained.

## Results

Eleven burn cases, (six males and five females)were enrolled into the study. Eight cases were caused by fire and three cases were caused by hot liquids. Two were intentional (self-immolation). Most burns (10 cases) occurred at homes.

For each burn case, one Haddon’s Matrix was prepared. Eventually, nine matrices were filled (four cases of burns in two of the same incidents had occurred). These matrices were then summarized into one whole Haddon’s Matrix. Two-hundred- and two affective factors were detected (See Table 1).

**Table 1:** Frequency distribution of possible of factors extracted from coded texts of the interviews in the 12 cells of Haddon’s Matrix.

*	Human	Appliance	Physical environment	Social (economic) environment	Total
Pre-event phase	14	21	20	25	80
Event phase	21	16	20	14	71
Post-event phase	19	1	21	10	51
Total	54	38	61	49	202

The pre-event phase, with 80 factors had the highest frequency compared with the other two phases in matrix rows. The physical environment column had the highest frequency with 61 factors compared with the other three columns.

Integrating the matrixes resulted in 43 main causes.“Human factor” in the post – event phase, with seven main factors had the highest frequency related to burn fatality. An appliance and physical environment in the post – event phase had the lowest frequency of factors. Based on the interviews in this qualitative research, the final Haddon’s Matrix, detailing possible factors that may cause fatal burns, is presented in Table 2.

**Table 2:** The affective factors in the incidence of burn mortality, based on phases and agents.

*	Human	Appliance	Physical environment	Social (economic) environment
Pre-event phase	- Lack of child and elderly care	- Non safe use of electrical and oil appliances (improper wiring, improper use of fire distribution)	- The non-safe building (being wood)	- economic problems (mostly regarding self-immolation)
	- Did not meet safety announcements	- Improper arrangement of electrical and oil appliances especially in the kitchen	- Unsafe carpeting in cooking/heating areas (Fig.1)	- Use of Unsafe appliance due to economic problems (Aladdin, picnic, etc.)
	- Lack of knowledge about burns prevention guidelines	- Use of hand-made unsafe appliances (Fig.1)		
		- not available to fire extinguishers capsule		
Event phase	- Age (age at above and below)	- Improper clothing (Pashmi and woolen clothes)	- Inadequate strength of wooden houses	- Wooden houses
	- Low level of knowledge on how to manage a burn event	- Use of improper appliances (flame distribution on the water heater, etc.)	- Being in hazardous environments (kitchen, bath, engine room, etc.)	- Keep flammable materials in and around the house (straw, a large quilt)
	- Lack of maintain vigilance and calming of other people	- Improper placement of Samovar (above dresser)		- Time (night)
	- Running on open air			
Post-event phase	- Low level of knowledge to deal with one burnt	- Unavailability suitable materials to deal with a burned (ointment, cold water and immediately after the burn.)	- being away from hospitals	- Lack of trust to staff and health system
	- Delays in getting the burned person to the hospital	- Improper transportation (Outdoor vans)	- Improper houses environment for the care of burnt	- Improper care at home
	- Failure to call the EMS or emergency medical			- High cost of health care and medications
	- Not referring to a health house or rural health center			- Poor services delivery in hospitals (Lacking a burn ward)
	- Improper transportation of a burnt			- Lack of appropriate emergency services to rural
	- Poor performance and inadequate skills of hospital staff			
	- Discharge Against Medical Advice(DAM)			

**Figure 1:**
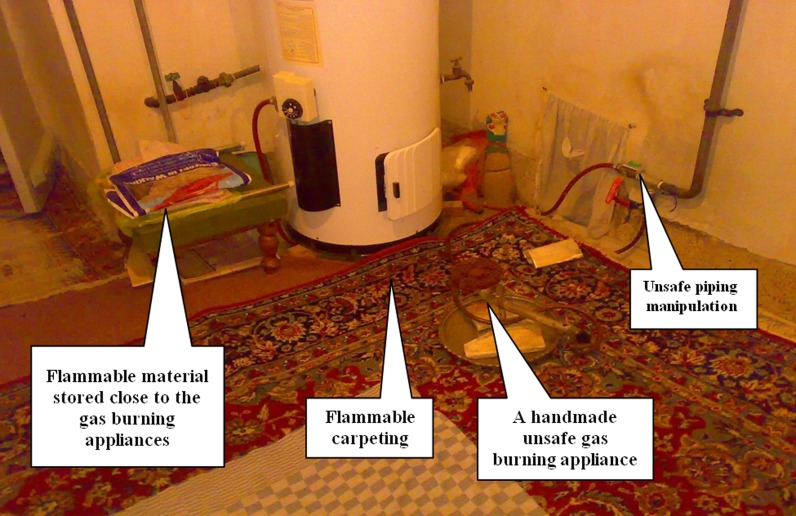
An example of unsafe appliances in hazardous environment captured during the study field survey.

Low levels of knowledge on how to manage a burn event in the event phase was one of the most important causes of fatal burns expressed by the participants. One said, "...yes, now I have learned that cold water should be used to cool the burn[wound], but we didn’t do it at that time…"

A major cause of self-immolation was reported to be economic problems. For example, one participants stated," What else he could do? He earned only 150 thousand Tomans (about $200) per month and it was intolerable to keep living with such a low income…" 

Poor service delivery in hospitals was reported to be one of the causes of burn fatality. In this context, one participant stated" My Dad didn’t receive good service at hospital, and you ask me why he has died?"

The unsafe and improper use of appliances such as Samovars and water heaters was another major cause of fatal burns. The following illustrate this theme" … No, Samovar was on the dresser…" Similarly another participant stated" … we took its (water heater) flame distributor and used it to warm up the house…"

## Discussion

Effective prevention strategies should focus on the causes of burns considering the setting in which such events occurs.^8^ Haddon’s Matrix can be a very useful tool. It has helped guide research and the design of interventions. Haddon’s Matrix was presented in the field of injury epidemiology in 1970 by William Haddon. Since that time, the matrix has been used as a tool to identify factors and to implement interventions for preventing many types of injuries. ^19^

Haddon’s Matrix is applicable in qualitative research in data collection and data analysis.^12^ In this study, we used Haddon’s Matrix to explore the possible causes of fatal burns in Ardabil Province. Through integration of matrixes 43 main causes were identified. The most common were: low level of knowledge on how to manage a burn event, the use of unsafe appliances in hazardous environments such as in the kitchen and bathroom, poor services delivery in hospitals (which often lack a burn ward), living in unsafe houses, economic hardships(mostly in the context of self-immolation) and other factors that detailed in Table 2).

The results of this study indicate that the unsafe use of hazardous appliances such as picnic, and water heaters, Aladdin, and Samovar indoors is one possible causes of mortal burns. This finding is consistent with those of Arshi and colleagues^8^ who found out that the majority of scald burns occurred during the use of heating appliances such as Samovars, gas stoves, Valors and picnic gas stoves. Moreover, the results of this study showed that the improper placement of Samovar and cooking appliances is one possible cause of fatal burns. This corroborates the findings of many previous studies in this field. ^20^ Our findings show that unsafe behaviors using Samovars which have an unstable base and placed on top of a dresser, (which may be accessible to preschool children) were one of the most common causes of fatal burns. Previous studies have shown that the unsafe use of Samovar is a determinant of burn injuries.^8,21^

In this study, a low level of knowledge on how to manage a burn event was one of the main causes of fatal burns. For example, in one of case involving a child, parents washed the burn area with warm water. In another the injured person ran out in to the open air. Peck and colleagues^22^ used Haddon’s Matrix to show that low levels of knowledge in people who reside in low and middle income countries is one of the predisposing predictors in the incidence and resultant complications in many burn cases. Mondozzi and Harper suggested that burn and fire educators can play a crucial role in helping families prevent these injuries. ^23^

The results of the present study show that developing educational programs that target women and children may prove effective. Sadeghi-Bazargani and colleagues^24^ who conducted their study in the same region, have emphasized this issue. Poor service delivery in hospitals and inadequate personnel skills were also revealed in this study. Dadkhah and colleagues^25^ in their study in Ardabil Province, consistently showed that the quality of services provided to burn patients were poor. The quality of hospital services have been identified by a few other studies is playing a significant role on burn mortality.^26,27^

Similarly a few previous studies found that financial problems (mostly in the context of self-immolation) affect the burn mortality.^28,29^ Therefore, attempts to reduce financial burden may be effective in reduction of mortality of burns due to self-immolation.

As with other qualitative studies, generalizability cannot be demonstrated and is not addressed in the present study. Due to different economic, social and cultural conditions, this study did not seek to generalize the results to all other conditions or settings.

## Conclusion

The results of this study indicate that low levels of knowledge on how to manage a burn event, the use of unsafe appliances such as Aladdin in hazardous environments including the kitchen and bathroom, poor services delivery in hospitals (which may lack a standard burn ward), living in unsafe houses and financial problems are most common factors in burn morality. These findings suggest several courses of action for the prevention and reduction of morality burns. These include: improving house and appliance-related safety, providing mass education, improving the quality of services in hospitals, implementing adequately equipped burn units, and improving the economic state of people. The use of Haddon’s Matrix is recommended in future injury research.
